# Hepatocellular carcinoma in Hepatitis C genotype 4 after viral clearance and in absence of cirrhosis: two case reports

**DOI:** 10.4076/1757-1626-2-7927

**Published:** 2009-09-15

**Authors:** Moutaz Derbala, Aliaa Amer

**Affiliations:** 1 Department of Gastroenterology & Hepatology, Hamad Medical Corporation, Doha, PO Box #3050, Qatar; 2 Department of Medicine, Weill Cornell Medical College, Qatar; 3 Department of Laboratory Medicine and Pathology, Hamad Medical Corporation, Doha, PO Box #3050, Qatar

## Abstract

Genotype 4 Hepatitis C virus represents approximately 20% of global Hepatitis C virus infection and is the source of a considerable burden to health-care providers across the globe. Many studies reported that interferon reduces the risk of hepatocellular carcinoma in patients with chronic hepatitis C.

Hereby, we are reporting two cases of hepatocellular carcinoma in Hepatitis C virus-genotype 4 after complete viral eradication and in absence of cirrhosis. We aim to highlight the possible direct oncogenic effect of Hepatitis C virus-genotype 4, particularly with concomitant bilharzial infection and the importance of life-log follow up of these patients even in absence of cirrhosis.

## Introduction

Preliminary evidence suggests that genotype 4 Hepatitis C virus (HCV) infection may place patients at greater risk for hepatocellular carcinoma (HCC) than other HCV variants [1].

The precise mechanism by which HCV infection causes HCC is not known. The development of cirrhosis is usually the cause of HCC in HCV infection. It was reported that IFN treatment prevents the development of HCC in patients with HCV-related cirrhosis regardless the response status [2]. However, the preventive effect seems to be stronger among sustained responders [3]. Few reported cases of HCC were described after HCV eradication, but none in genotype 4. Most of reports suggested that old age, presence of cirrhosis and genotype 1 had the high risk of developing HCC after sustained response (SR) [4]. We are describing for the first time 2 cases, middle aged of genotype 4, who developed HCC after viral clearance in spite of pre-treatment low viral load and absence of cirrhosis.

We aim to highlight the possible direct oncogenic effect of HCV-genotype 4 and the importance of life-log follow up of these patients even in absence of cirrhosis. In addition, we shall discuss the impact of concomitant schistosomal co-infection on progression to HCC.

## Case presentation

### Case report 1

A 47-year-old, Egyptian, male patient, was diagnosed to have post HCV chronic active hepatitis since 1995 and treated with Chinese herbal therapy. There was no history of blood transfusion, alcohol consumption or drug abuse. In 2006, he was scheduled in Hamad Hospital, Qatar for Pegylated-interferon (Peg-IFN) - Ribavirin therapy and pre-treatment investigations showed positive PCR for HCV-RNA with titer 328,000 IU/ml, genotype 4 and liver biopsy was of grade I/IV stage II/IV (according to Scheuer Score), minimal steatosis but no excess iron or malignant changes. Liver function tests (LFT) showed increased alanine amino-transferase (ALT) 107 U/L. He has thrombocytopenia 150,000 × 10^3^/ml. Sero-markers for concomitant Hepatitis B (HBV) were negative. Abdominal ultrasound scanning did not show focal lesion and Alfa-fetoprotein (AFP) was 34 IU/ml. He was treated between February 2006 and Feb 2007 with Peg-IFN 2a 180 ug weekly in combination with ribavirin 1000 mg daily. Because of thrombocytopenia since second dose, Peg-IFN2a was reduced to 135 ug till the end of treatment. Viral clearance was detected since week 4. At the end of 48 weeks-treatment, PCR was negative for HCV-RNA, ALT 51 U/L, Platelet 150,000, and AFP 31.3 U/ml.

In march 27, 2008 the he presented with right hypochodrial pain and low grade fever. Computed tomography (CT) abdomen showed an area of HCC within posterior t sector of the right lobe with predominant disease centered within segment VI and VII, evidence of tumor thrombus within the portal vein. No lymph node involvement was noted. The diagnosis was confirmed by liver biopsy. PCR was still negative for HCV-RNA. Local treatment chemo- embolization was performed on March 25, 2008, followed by orthotopic liver transplantation in May 5, 2008.

### Case report 2

A 48-year-old Egyptian male patient presented in 2000 with positive serology for HCV and high transaminases ALT/AST 182/120 U/L, while albumin and bilirubin were normal. He had history of bilharziasis treated with tartar emetic during childhood and with praziquantel in 1991. PCR was positive for HCV-RNA at a titer 104,000 IU/ml of genotype 4. CBC was normal (Platelet 115 × 10^3/ul and Hemoglobin 16.5 g/dl). Liver biopsy showed picture of interface hepatitis, with fibrosis of Stage III/IV (according to Scheuer Score), with no stainable iron, malignancy or steatosis detected. AFP was 23.1 IU/ml. Serological screening for concomitant HBV was negative. There was no alteration in serum iron balance. There was no history of alcohol consumption or drug abuse. He was treated with Pegylated-IFN2a 180 ug weekly and Ribavirin 1200 mg daily for 48 weeks with complete sustained response. He showed rapid virological response (RVR) with viral clearance at week 4. Last dose was in February 2003. Follow up LFT showed biochemical response (although it was high during treatment), normal blood picture, normal thyroid function, normal coagulation profile, and AFP of 20 IU/ml. Annual follow up showed sustained virological response till May 2007. After 4 years of sustained virologic response, the patient was referred to a hospital and Ultrasound scanning revealed a hepatic focal lesion, which was confirmed MRI, which also showed a suprarenal focal lesion. Right hepatic lobe focal lesion measured 11.8 × 11 cm. The mass was beyond surgical resection and criteria for liver transplantation, so the patient received local palliative treatment with chemo-embolization. At that time AFP was 42 IU/ml. Follow up after 6 sessions of local treatment showed minimal decrease in the largest lesion and increase in necrotic component with decreased in enhancement in all liver lesions. AFP was 50 IU/ml. PCR for HCV is still negative till now.

## Discussion

Unlike HBV, HCV does not integrate into the host genome [5]. Accordingly, the development of cirrhosis seems to be the major risk factor for HCC in HCV infection. It is reported that, viral clearance is associated with improvement in liver histology, stabilization of liver disease, and possibly, reduction in risk of HCC [6]. In our cases and previous reports [7,8], the patients developed HCC in spite of viral clearance, suggesting that HCV per se could have a direct oncogenic effect. This may be attributed to genetic mutation induced by HCV. Consistent differences in gene expression patterns were recently reported in HCV-HCC compared with early HCV cirrhosis, late HCV cirrhosis, and normal controls [9]. Experimental observations suggest that certain HCV genomic regions, such as Core and non-structural protein-3 (NS3), might have oncogenic properties [10]. Recent studies reported a relationship between the presence of mutations within the IFN-sensitive determining region (ISDR) and the development of HCC. HCV-genotype 4 has heterogeneity within the hypervariable region 1 and the NH2 region of the E2 protein. These indicate that HCV genotype 4 in Egypt is extremely variable not only in terms of sequence, but also in terms of functional and immunologic determinants [11]. Genotype 4 infection may be considered a risk factor for neu-oncoprotein over-expression and subsequent development of HCC [12]. Also, HCV RNA was detected in the serum, liver, and tumor tissues of patients with HCC [13]. It is also worth mentioning that in Japan, the development of HCC in patients without cirrhosis is routinely reported, even with mild degrees of fibrosis [14]. Similarly, case 1 presents progression to HCC in a case of minimal fibrosis, suggesting that progression to HCC may occur in absence of cirrhosis and possibly providing further evidence for the direct oncogenic effect of HCV. IFN itself, through its growth-inhibitory effect, may delay the growth of HCC [15]. Therefore, the actual mean time for the tumor formation of HCC of 1 cm in diameter may be longer than 6 years [16], and it is reasonable to consider that almost all HCC detected within 10 years of the follow-up period after IFN therapy had already developed prior to IFN therapy. So, the late appearance of HCC in our case (more than 4 years) and previous cases (90 and 70 months) [7] may be attributed to the oncogenic effect of HCV prior to eradication, providing another substantial evidence.

The virological response persists over time and is associated with a very low incidence of liver complications. Advanced age both at inclusion, or at infection, viral genotype 1, low platelets, advanced histological staging and possibly some specific HLA alleles [15]-[16], are factors independently associated with a faster rate of progression towards liver complications. In our cases, all were middle aged and of genotype 4. From our cases and others, viral load and genotype did not seem to influence the progression to HCC. An important point which still needs to be clarified is whether viral eradication tested in our patients and others reflects a real clearance from hepatic tissue? None of reported cases checked PCR in hepatic tissues in spite of the fact that early report by Esaki et al 2004, suggested that HCV-RNA was detected in liver tissue in HCC patient with negative HCV serology [17]. Also, recent studies, have found trace HCV viral material in liver tissue both among sustained responders and in patients with chronic liver disease who were HCV RNA negative, suggesting occult hepatitis C [18]. Within the past few year, published reports have strongly proposed the notion that HCV RNA may be expressed in liver and extrahepatic reservoirs, in the absence of circulating viremia, and that the virus may be active, and may facilitated the development of HCC [19].

In our opinion, liver tissue HCV-RNA seems to be an important tool in confirming viral clearance; however, the idea of using an invasive technique to follow up a patient who has presumably eradicated his virus might be not easily accepted.

Our patients have concomitant bilharzial infestation. Concurrent *Schistomsoma mansoni* infection may have an important impact on the outcome of liver disease in patients with HCV, with a higher incidence of cirrhosis and elevated risk for hepatocellular carcinoma seen in HCV genotype 4 and schistosomiasis coinfected patients than in those with HCV alone [20], histology scores for grade (total inflammatory score) and stage (fibrosis/cirrhosis score) were significantly higher in coinfected individuals.

We can conclude that HCC may developed in HCV genotype 4 after viral clearance because direct oncogenic effect through genetic mutation which may take place before starting interferon therapy, extrahepatic and possible occult HCV, and concomitant bilharzial infection. So, we recommend life-long follow up screening with ultrasound and alpha-fetoprotein after HCV clearance in genotype 4, should be considered for all cases, regardless the stage of fibrosis or presence of concomitant factors for progression to HCC.

## Consent

Written informed consent was obtained from both the patients for publication of this case reports. A copy of the written consent is available for review by the Editor-in-Chief of this journal.

## Competing interests

The authors declare that they have no competing interests.

## Authors' contributions

MD provided clinical care to the patient, wrote the manuscript and revised the manuscript. AM analyzed and interpreted the data regarding hematological and other laboratory findings, involved in the design and preparation of the manuscript. All authors read and approved the final manuscript.
